# A Scalable Optoelectronic Neural Probe Architecture With Self-Diagnostic Capability

**DOI:** 10.1109/TCSI.2018.2792219

**Published:** 2018-01-24

**Authors:** Hubin Zhao, Ahmed Soltan, Pleun Maaskant, Na Dong, Xiaohan Sun, Patrick Degenaar

**Affiliations:** 1 Newcastle UniversityNewcastle upon TyneNE1 7RUU.K.; 2 University College LondonLondonWC1E 6BTU.K.; 3 School of Electrical and Electronic EngineeringNewcastle UniversityNewcastle upon TyneNE1 7RUU.K.; 4 Tyndall National InstituteUniversity College CorkT12 R5CPCorkIreland; 5 South East UniversityNanjing210018China

**Keywords:** Active optrode, neural stimulation, optogenetics, implantable, self-diagnostic

## Abstract

There is a growing demand for the development of new types of implantable optoelectronics to support both basic neuroscience and optogenetic treatments for neurological disorders. Target specification requirements include multi-site optical stimulation, programmable radiance profile, safe operation, and miniaturization. It is also preferable to have a simple serial interface rather than large numbers of control lines. This paper demonstrates an optrode structure comprising of a standard complementary metal-oxide-semiconductor process with 18 optical stimulation drivers. Furthermore, diagnostic sensing circuitry is incorporated to determine the long-term functionality of the photonic elements. A digital control system is incorporated to allow independent multisite control and serial communication with external control units.

## I. Introduction

The optogenetic technique holds a remarkable potential for the realization of advanced neuroprostheses. By genetically expressing light-sensitive proteins such as channelrhodopsin-2 (ChR2) in cell membranes, targeted neurons in the brain or other neural tissue can be controlled by light [1]. This neuromodulation technique can be applied to both the exploration of extensive brain networks and potentially to provide effective therapies for neurological disorders.

For optogenetic stimulation, precise, intense, light delivery is an essential requirement. Classically, ChR2 requires a blue (470 nm) optical irradiance of ~1 mW/mm^2^ [2], [3]. Other variants are now available with lower thresholds and different wavelengths [4]. However, it is still the case that light scatters strongly in neural tissue. As such, light must either be delivered locally through a light-guide or generated locally to the tissue [5]. Each case has advantages and disadvantages.

Light guides [6]–[8] can be highly biocompatible, and as passive structures, do not cause heating of the brain tissue. Micro-fabricated probes with multiple light guiding structures have been successfully demonstrated since 2011 [9]–[12]. However, it is challenging to achieve efficient optical coupling and multiplexing. Furthermore, it is challenging to emit light at right angles to the probe. Thus, emitted light tends to traverse cortical layers rather than transmit through specific layers.

In contrast, if probes have local emissive elements, then multiplexing can be achieved electronically. Furthermore, radiance from emitters is typically perpendicular to the probe and will thus traverse along rather than through cortical layers. The caveat is that the emitters need to be efficient to ensure there is no negative tissue heating. Additionally, the overall design is more complex. Currently, micro-scale lasers do not function efficiently in the blue-green region of the optical spectrum. As such, the primarily available emitter class is gallium nitride-based (mini or micro) Light Emitting Diodes (LED) [13]. The primary difference between mini and micro LEDs is that the former is commercially available in dimensions down to $200~\mu \text{m}$ and can readily be bonded onto probes. The latter can be custom fabricated to dimensions of 20 $\mu \text{m}$. The former is suitable for broader area emission and the latter for higher resolutions.

In 2013, Cao et al. demonstrated a probe which could record and emit light [14] via a single mini ($200\,\,\mu \text{m}\,\,\times600\,\,\mu \text{m}\,\,\times $ 1000 $\mu \text{m}$) LED. In 2013, McAlinden et al. demonstrated an optrode fabricated directly from Gallium Nitride (GaN), allowing multiple micro LEDs to be integrated onto the surface [15]. In the same year, Kim et al. proposed a multifunctional neural probe with GaN LEDs [16]. More recently micro LEDs transferred onto diamond [17] and silicon substrates have been demonstrated [18]. Recently Biederman et al. [19] demonstrated a control system which could externally multiplex and control emission. We have also previously demonstrated control electronics for 2D arrays of high radiance light emitters [13], [20]. However, electronic control, integrated within a probe has not yet been demonstrated.

In contrast in the field of electronic probes, a number of highly integrated probes exist which can record from a large number of electrodes simultaneously. Shulyzki et al. [21] have demonstrated a chip, which can be combined with existing 8 $\times $ 8 Utah style probes and up to 256 external recording pads. More interestingly Lopez et al. [22], and Angotzi et al. [23] have both demonstrated probes fabricated from a CMOS base which incorporates 455 and 512 recording electrodes respectively. These architectures utilize the shaft area for active electronics, which allows for much larger scale integration. But, these are purely electronic, and cannot be used for optogenetic applications.

**Fig. 1.**
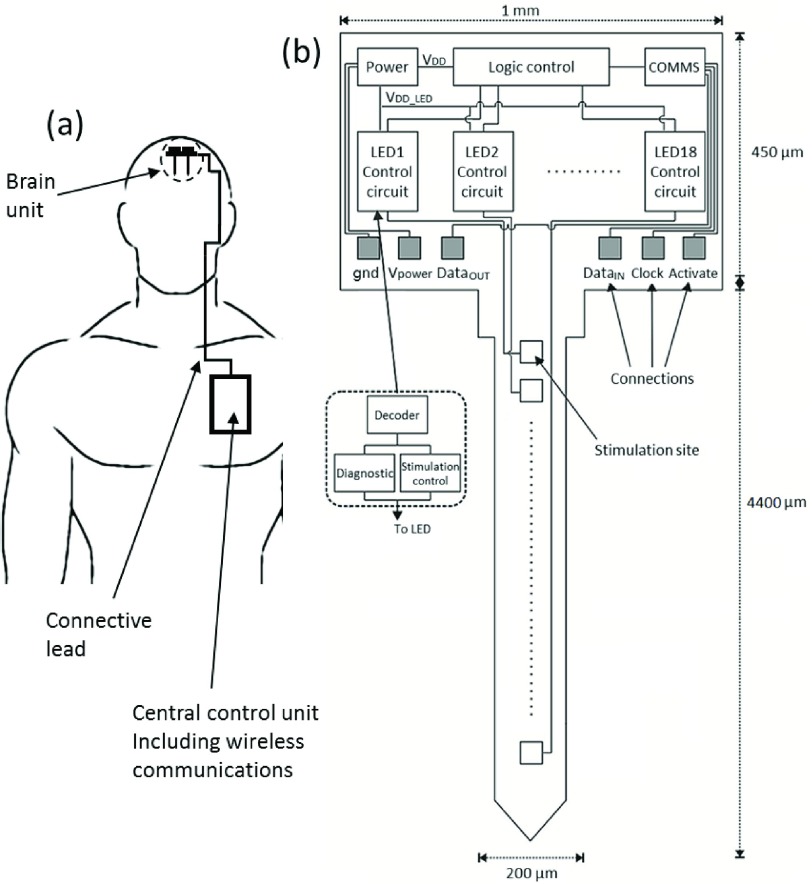
The system architecture of proposed optical probe. (a) A biomedical use of such a probe with a central control unit communicating over a digital communication method to the optrodes forming a brain implant. (b) The primary components of the optrode including communications, logic control and LED driver circuitry.

In this work, we propose to progress the concept of CMOS based active optoelectronic probes for the field of optogenetics. To our knowledge, this submitted work is the first scalable CMOS-based active optogenetic implant. It is possible to utilize the same post-fabrication techniques as for other probes to shape our probes and host sites for light emission and electrical recording. Our probes are designed to host GaN $\mu $LEDs. A conceptual diagram in Figure 1 demonstrates how this optrode would be utilized. A brain unit comprising of such probes would be controlled by an embedded control unit with digital communications between the two probes. Figure 1(a) demonstrates this for medical intervention. Figure 1(b) shows the key components we propose in this probe. We have previously [24], [25] demonstrated low-density optrode designs with consisting of $6\times $LEDs. These were designed to use high efficiency mini-LEDs for brain pacemaking activity. In contrast, this paper seeks to explore probe architectures which can scale to a much higher density array, while still allowing for individual intensity and PWM control of individual LEDs. Depending on requirements, recording electronics as per Lopez et al. [22], and Angotzi et al. [23] could also be incorporated for closed-loop applications.

Moreover, since the optrode (including the LEDs) is intended to be implanted into brain tissue, the system stability is a key concern for long-term chronic operation. There is little data about how active probes would perform under such conditions. One hazard is that optrode may be damaged during or after implantation. Post-implantation, there may be corrosion of the individual light emitters. Since it would be challenging to evaluate the operation statuses of the optrode from external medical imaging, we have incorporated diagnostic sensing circuitry to assess the continued functional state of the optrode.

## II. System Architecture

Our circuitry has been developed using a standard 0.35 $\mu \text{m}$ CMOS process from X-FAB. The floorplan is shown in Figure 1(b). The optrode design consists of two primary parts: the optrode head in which all of the active circuitry is included, and the CMOS/silicon-based optrode shaft.

The length of the optrode shaft has been set to 4.4 mm, to match the thickness of primate brain cortical layers (which vary between 2 mm and 4.4 mm) [26]. As the cortex has up to six layers depending on the region, we have included six banks of individually controllable micro-photonic emitters, each with clusters of three micro-photonic emitters. Such clustering may make placement more convenient. The width of the shaft can be variable depending on LED size, but we assume a minimum $200~\mu \text{m}$ for structural rigidity.

Eighteen LED control modules have been generated in the optrode head to access the individual LED contact point. Each LED control module can perform both stimulation control function and diagnostic sensing function. Moreover, in order to address and control the whole system, a global communication and logic control module is implemented in the head part. This determines all of the input/output data of the optical stimulation circuitry and the diagnostic sensing circuitry. Subsequently, each LED point can be individually controlled and sensed. In addition, six I/O pads are implemented to either convey input/output signals or provide power supplies.

## III. Control System

**Fig. 2.**
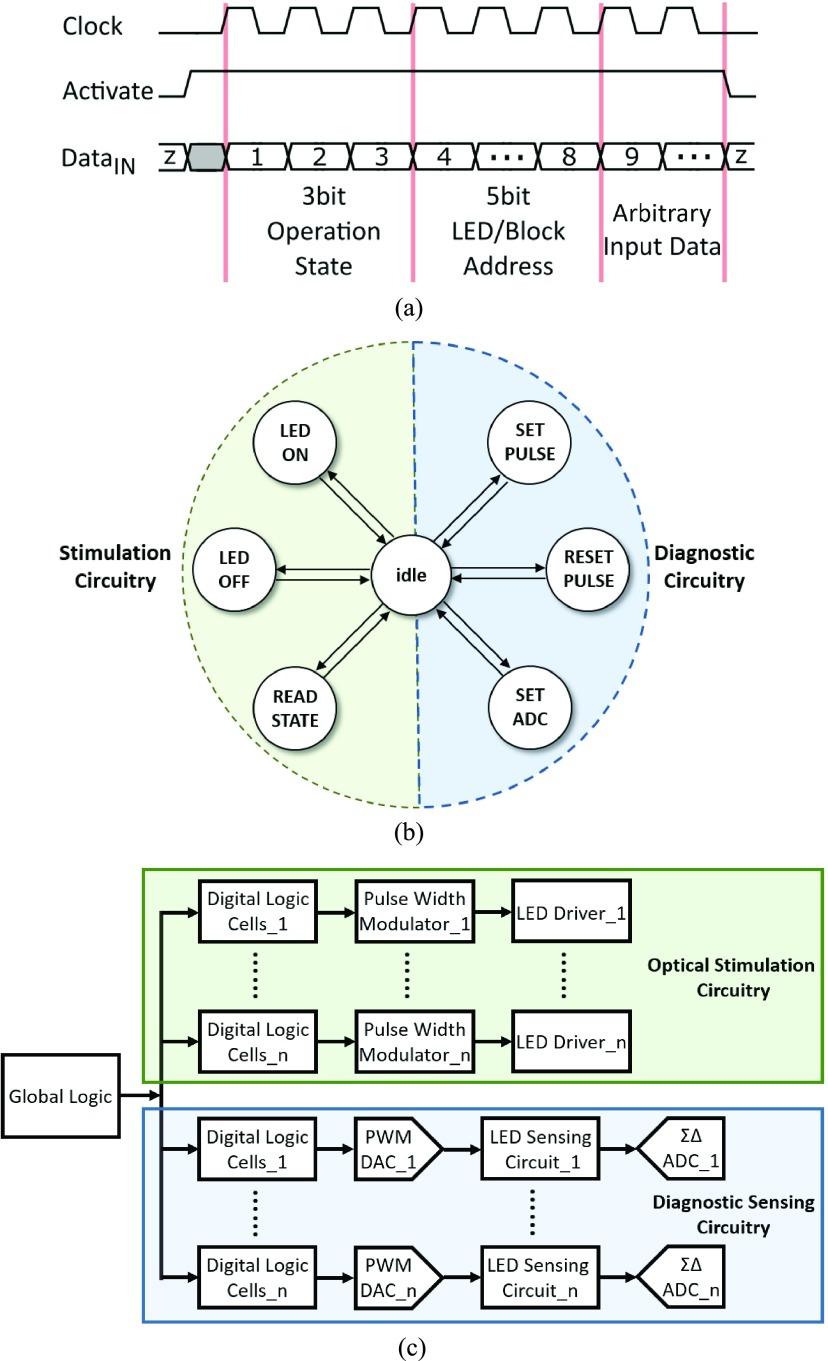
(a) Timing diagram of the modified SPI protocol. (b) Command trees of the FSM states and corresponding transition conditions. (c) Flowchart of the overall system, including logic control, optical stimulation circuitry, and diagnostic sensing circuitry.

Figure 2 demonstrates the logic control subsystem. A modified serial peripheral interface (SPI) protocol has been developed for duplex communication, and the corresponding timing diagram is depicted in Figure 2(a). A global clock signal (*Clock*) is defined to synchronize all the logical operations of the optrode. An external activate (*Activate*) signal determines the state of a particular operation. A finite state machine (FSM) has been implemented to master all the operation states. All FSM states and corresponding transition conditions are illustrated in Figure 2(b). *LED* $_{ON}$ and *LED* $_{OFF}$ are two major working patterns for the stimulation circuitry, and these two modes can manipulate the micro-photonic emitters with a predefined pulse width. The *Read* function is used to monitor the real-time status of the micro-photonic emitter via the pulse width modulator. *Set_Pulse* and *Reset_Pulse* are two input signals for the pulse modulated Digital-to-Analog Converter (DAC), which can achieve the voltage scanning scheme for diagnostic purposes. The *Set_ADC* is a trigger signal to enable or disable the Sigma-Delta Analog-to-Digital Converter ($\Sigma \Delta $ ADC). The $\Sigma \Delta $ ADC could then record the diagnostic sensing voltage, and the recorded data is then fetched via pulse expressions. Once the operation mode is decided, the targeted micro-photonic emitter would be consequently defined by LED addressing module. Then, the active micro-photonic emitter, *LED_n_*, is selected by decoding the address information from input data *Data* $_{IN}$. The flowchart of the overall system is displayed in Figure 2(c).

## IV. Optical Stimulation Circuitry

The neuron response will vary with the integral function of the irradiant density within short time periods [27], [28]. Thus, within a defined short period (frame) both pulse width modulation at a maximum amplitude or amplitude modulation are equally valid.

### A. Light Requirement

There is a considerable body of work exploring the light requirement for optogenetic cells. The original 2003 paper [2] described the threshold as 0.7 mW/mm^2^. This threshold was confirmed for dissociated neural culture, e.g. by Han et al. [29]. Since then new types of opsins and in-vivo data have shown varying requirements, but the currently accepted value within the community is 1 mW/mm^2^ to achieve strong optical control.

**Fig. 3.**
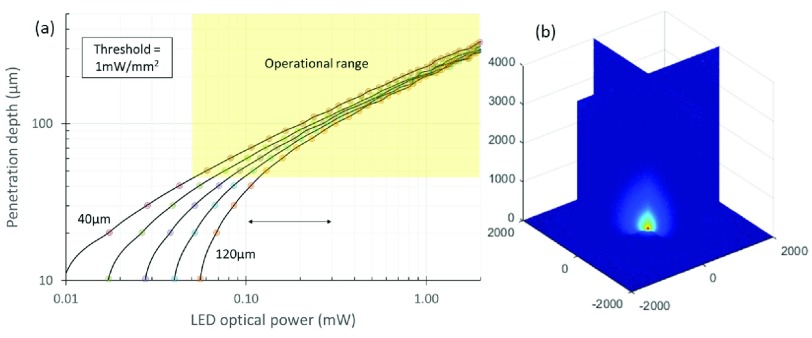
Light transmission in tissue (a) the optical power required for a Lambertian emitting LED to reach a certain penetration depth for a 1 mW/mm^2^ threshold. This is given for different emission region diameters from $40~\mu \text{m}$ to $120~\mu \text{m}$. (b) the 3D profile of light penetration through tissue.

Wu et al. [18] and McAlinden et al. [15] have previously demonstrated modelling of light scattering behavior in neural tissue. In order to understand what this means in terms of the light emission, we have replicated this work for a LED emitting with a typical Lambertian (intensity varies with the cosine of the emission angle) profile. Results can be seen in Figure 3.

The light requirement depends on penetration depth as well as LED size. Plotting in this form, it is possible to determine the required absolute radiance (and thus required current) for a given LED diameter to reach a given penetration depth. We highlight the operational range given the circuit and LED efficiency parameters below.

To compare with previous literature, Wu et al. [18] developed non-electronic probes with built-in LEDs. They used LED currents of $45~\mu \text{W}$ to generate $8~\mu \text{W}$ of optical power. They used modelling to estimate penetration depths of around $60~\mu \text{m}$ at the 1 mW/mm^2^ target irradiance. This is more optimistic than our modelling shown in Figure 3. Nevertheless, they demonstrated the ability to stimulate nearby neurons. Another example is by McAlinden et al. [15] who utilized currents of up to 5 mA to generate radiances of 0.6 mW. The resultant penetration depth at the target irradiance was 175 $\mu \text{m}$ and matches our modelling in Figure 3.

The target range for this work is to achieve $100~\mu \text{m}$ at an irradiance of 1 mW/mm^2^. It should be noted that we expect to achieve greater depths in practice. For example, Wu et al. saw in-vivo responses at expected irradiances of 0.1 mW/mm^2^.

### B. LED Characteristics

The design has been developed to utilize either custom fabricated $\mu $LEDs or mini-LEDs from commercial sources. In order to characterise the $I$-$V$ and *I-L* (*Luminance*) relationships, we explored operation with both a $20~\mu \text{m}$ diameter $\mu $LED custom fabricated at the Tyndall Institute [30], [31], as well as a commercial surface mountable LNJ947W8CRA mini-LED from Panasonic ($200~\mu \text{m}$ diameter).

**Fig. 4.**
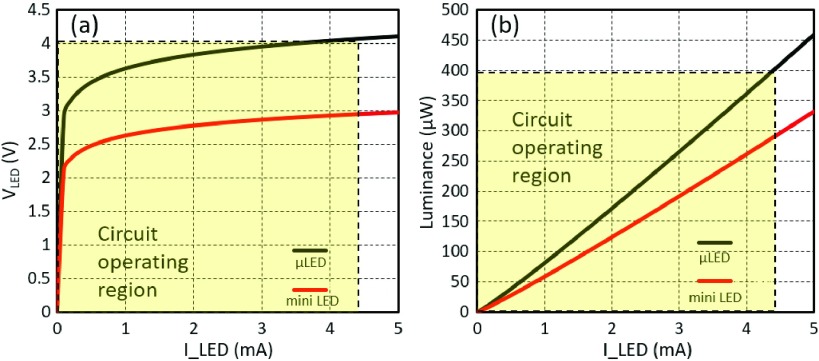
*I-V-L* relationships of the $\mu $LED and the commercial Panasonic mini LED. The current through each LED is steadily increased from 0 mA to 5 mA. (a) *I-V* relationship. (b) *I-L* relationship. The efficiency measurement of both LEDs is around 3% which is consistent with the literature.

*I-V-L* measurements were taken using the same approach described in the experimental section below, and a Verilog-A model was derived to inform our simulations. Based on the optical experiments, the $I$-$V$-$L$ relationships of both $\mu $LED and mini LED are illustrated in Figure 4. Moreover, we measured efficiencies for both LEDs at ~3%. The operating region of the circuit is shown in dotted lines. Although the global voltage $\text{V}_{DD\_{}LED} $ is 5 V, 1 V is consumed by the $\text{V}_{DS}$ of the drive transistor, thus limiting the output current, and therefore radiance.

The measured efficiency was consistent with what has been provided in the literature to date for $\mu $LEDs in the optrode literature ([14], [15], [18]). In the long term, other groups developing mini and micro-LED architectures for other purposes have demonstrated have significantly better wall-plug efficiencies. For example, the CREE DA2432 mini LED [32] has efficiencies exceeding 30%. This is clearly a much larger LED ($320 \times 240 \,\,\mu \text{m}^{2}$), and thus not scalable to high integration. But Narukawa et al. [33] have demonstrated driving LEDs at similar current densities to micro-LEDs whilst still achieving 35% efficiency. As such, we believe that further progress to reduce series resistances and improve efficiency is possible in the micro-LED field.

An important final point to note is the heat emission from LEDs at the current and voltage levels above can cause heating of the probe. If only 3% of the input energy is converted to light, 97% is therefore converted to heat, limiting the ON time within for a given maximum surface temperature.

As the current, voltage and efficiency parameters are similar to that of McAlinden [15], we point to their study of the subsequent thermal effects on their passive probe (i.e. without circuitry). They estimate that the LED can be driven for up to 100 ms while seeing a maximum temperature rise of 1 °C over the 37 °C ambient.

### C. LED Driver

For blue LEDs, we expect an operational range between 3.5 and 4 V to achieve a target current of up to 4 mA. With a 1 V drop across the drive transistor, a 4 V operation is required. In the long term, if LED efficiencies are improved, the required current can come down. Additionally, if contact resistances are improved, the required voltage may be reduced to 4.5 V or perhaps slightly lower. If red LEDs are used in conjunction with red-shifted opsins, it would be theoretically feasible, assuming exceptional efficiencies, to bring the voltage down to within a 3.3 V domain. Thus, in this design, a 5 V pMOS transistor is utilized in the LED drive circuit. Given the trade-off between driving ability and dimensions, the $W/L$ size of the pMOS drive transistor is set as $160~\mu \text{m}/1~\mu \text{m}$. With a $V_{DD\_{}LED} = 5$ V, the maximum $\mu $LED driving current is 4.4 mA. The maximum radiance is 400 $\mu \text{W}$, which is sufficient for targeted local light delivery. Monte Carlo analysis was performed on the driver circuit indicating a standard deviation of $54~\mu \text{A}$ or ~1%.

**Fig. 5.**
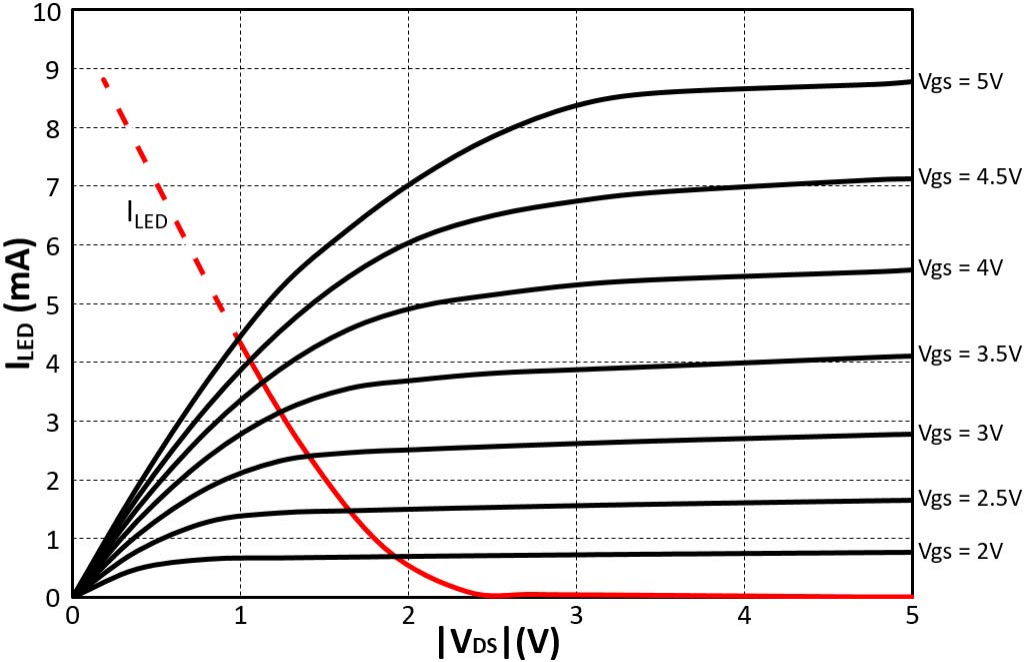
Load line plot for the drive transistor and the $\mu $LED. When $V_{gs} = 5$ V, the maximum value of $I_{LED}$ is 4.4 mA, and corresponding utmost luminance is $400~\mu \text{W}$.

Figure 5 shows a load line plot for the drive transistor and the $\mu $LED. For much of the operation, the transistor will be in the triode region and thus acts more like a variable resistor than an ideal current source. This has a number of implications: to obtain ideal current driving in the saturation region would require a larger $\text{V}_{DD\_{}LED}$ i.e. $V_{DS}=V_{DD\_{}LED}-V_{LED}>V_{GS}-V_{T}$, thus: $V_{DD\_{}LED}>V_{GS}-V_{T}+V_{LED}$, where $V_{LED}$ is voltage across the LED which is typically in the range 2.5 – 4 V depending on drive current. Such additional voltage would reduce the efficiency.

Operating the drive transistor in the triode region is more efficient as it minimizes the $V_{DS}$ drop across the drive transistor. However, the operational performance of LEDs bonded-on post fabrication will be more susceptible variability in contact resistances. As such, each LED would need to be individually calibrated post-fabrication.

The LEDs can be:

1) *ON supra-threshold* – i.e. forward bias beyond 2.6 V with light emission. Our circuit is designed to drive this at a set voltage oscillated in time through pulse width modulation, and also the intensity.
2) *ON sub-threshold* – i.e. forward bias below 2.6 V with no light emission. According to Cao et al. [34] and Xi et al. [35], the LED leakage operates in a thermally assisted hopping manner in the first ~1 V which is the same as the reverse leakage. As such, it is possible to determine the operational characteristics of the diode in the first few volts.
3) *OFF* – i.e. in grounded state (and of course no light emission)
4) *REVERSE bias* – not implemented on this design. But theoretically, there may be an advantage in biphasic operation – matching the positive phase with the negative phase from a degradation perspective [36]. However, it would also complicate the design as each LED would then need an individual cathode– reducing the potential integration density.

### D. Pulse Width Modulator

The most efficient driving scheme for the LEDs in our design is pulse width modulation (PWM) at maximum intensity - assuming the droop profile of the LED (i.e. how the LED efficiency drops with increasing current) is low. This is because, with intensity modulation, efficiency decreases with decreasing light intensity because of the voltage drop across the drive transistor. For eight bits of intensity modulation within a typical frame time of 20 ms, the minimum required ON/OFF switching cycle is $78~\mu \text{s}$.

**Fig. 6.**
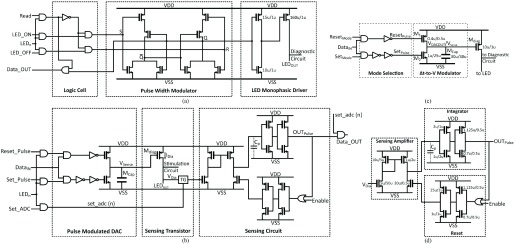
(a) Circuit schematic of stimulation control block. $LED_{ON}/LED_{OFF}$ signals determine the operation status of emitters. *Read* signal monitors the real-time working state saved by PWM unit. (b) A circuit schematic of diagnostic sensing block. *Set_Pulse* and *Reset_Pulse* generate pulse signals to achieve voltage scanning. *Set_ADC* determines the timing control of the $\Sigma \Delta $ ADC to fetch the $V_{Dia}$. (TG is short for transmission gate). (c) Circuit schematic of the pulse width modulated DAC. This DAC performs two operations: mode selection and time-to-voltage conversion. (d) The diagnostic sensing element. Left is a (non-OTA) transconductance amplifier which feeds current into a simple $\Sigma \Delta $ ADC (right). This consists of three stages: current buffering, integration, and reset/pulse control.

Each optical stimulation block has individual active logic circuitry to allow simultaneous operation of all LEDs. In order to pulse LED operation, the LED operation status (‘*ON*’ and ‘*OFF*’) needs to be maintained and updated in real time with particular stimulation cycles. The pulse width modulator keeps this working state constantly to maintain stimulation cycle until new data is pushed in and the new working mode is updated. A SR latch is used as a memory cell to maintain the ON state, as shown in Figure 6(a). In a post-layout simulation, the maximum frequency of the standalone SR cell is 125 MHz. Then this design is connected to the LED driver. The maximum switching frequency is recorded as 30 MHz which is significantly beyond the 50-$100~\mu \text{s}$ minimum switching time requirement. The design was calculated to consume 63.9 fJ energy per cycle.

## V. Diagnostic Sensing Circuitry

### A. Local Diagnostic Block

When the optrode is performing a normal light emission operation, the drive current through the LED will have a diode behavior with applied $V_{DD\_{}LED}$ until limited by the control transistor. There may, however, be an abnormality due to failure in the passivation, corrosion of LED contacts or even a critical failure such as probe fracture during insertion.

Electronically such scenarios may be seen as a resistance (corrosion in contacts or leak to tissue, or leak between the contacts). Should such processes continue, electrolytic degradation of the contact could lead to open circuit behavior (complete corrosion). An open circuit could also represent probe fracture. We believe these scenarios to be the primary route to degradation. There is an additional scenario whereby there is a rapid delamination of the encapsulation between the anode and cathode, e.g. this could happen between infrequent diagnostic sensing. This would look like a direct resistance between the anode and cathode– in parallel with the LED.

It is thus possible to analyze such effects (if they exist) by performing a DC voltage scan across a given LED and then determining the current-voltage profile. The local diagnostic sensing block consists of three different functioning modules (Figure 6(b)): a pulse-width modulated DAC, a source transistor $M_{Diag}$, and a sensing circuit. The latter consists of a low-gain transconductance amplifier and a one-bit $\Sigma \Delta $ ADC. The transistor sizing of the sensing block is given in Figure 6(d). The pulse-width modulated DAC (Described in $B$) determines the voltage $V_{Sense}$ and thus the $V_{gs}$ on $M_{Diag}$. This transistor is operated in strong inversion triode mode as most of $V_{DD}$ will drop across the LED.

In order to avoid turning on LED, $V_{Sense}$ is varied between 4.25 and 3.5 V, i.e. $\vert V_{gs}\vert =0.75$ - 1.5 V respectively. This is achieved by ensuring pulse widths are below 150 ns. Since the diagnostic function is expected to be operated without stimulating light emission, the $M_{diag}$ is set as 10 $\mu \text{m}$/1 $\mu \text{m}$ to limit the sensing current range into a safe region. The resulting sensing current ($I_{Dia}$) creates a voltage across $V_{Dia}$ in order to pass through the LED. As such, changes in the contact impedance at the anode or cathode will determine the profile of $V_{Dia}$ vs $V_{gs}$.

Ideally, the most accurate way to measure $V_{Dia}$ is to utilise a full operational/instrumentation amplifier. However, the objective here is to explore scalable solutions whereby each LED has its own sensing circuit. As such the sensing circuit described in Figure 6(d) comprises of a low-gain transconductance amplifier (i.e. “sense amplifier”) connected to a $\Sigma \Delta $ ADC module (described in section $C$). The transconductance amplifier charges up a parasitic capacitor in the integrator stage, and the signal is then reset by the Reset stage. As the $V_{Dia}$ has an inverse relationship with a time interval ($t_{interval}$) between an enable trigger and the output pulse, the $V_{Dia}$ can then be noted and reconstructed by recording the $t_{interval}$. The operational profile of this circuit is given in Table I.

**TABLE I:** Main Parameters of the Diagnostic Circuit

Technology	X-FAB 0.35-$\mu \text{m}$, 4M CMOS
Operation voltage	3.3 V/5 V
Sampling points	130/80
Pulse modulated DAC	
Dynamic range	43 dB/38 dB
Input voltage range	0 - 3.3 V/5 V
Output voltage range	0 - 3.3 V/5 V
Circuit area	$30\,\,\mu \text{m}\times 40\,\,\mu \text{m}$
Static power consumption	3.7 fW/5.77 fW
Dynamic power consumption	$3.34~\mu \text{W}/8.45~\mu \text{W}$
Energy per sequence	(max) 10.3 pJ/24.3 pJ
Clock frequency	10–100 MHz
$\Sigma \Delta $ADC	
Input voltage range	0.75 - 3.3 V/0.75 - 5 V
Out pulse resolution	10 ns
Dynamic range	100 dB
Circuit area	$30\,\,\mu \text{m}\times 27\,\,\mu \text{m}$
Static power consumption	1.6 fW/3.3 fW
Dynamic power consumption	$4.8~\mu \text{W}/12.6~\mu \text{W}$
Clock frequency	(max) 100 MHz

Although the gain of the transconductance amplifier is low, providing between 4:444 nA for the range of 1:5 V, it is sufficient for most of the scenarios presented. However, it cannot sense $V_{Dia} < 0.75$ V i.e. when the amplifier stops being in strong inversion. As such, should the anode-cathode voltage drop below this (for example if in the short circuit case), then the output of the sensing amplifier will drop to zero. The output of the $\Sigma \Delta $ ADC will then timeout. If this is the case, although it cannot be characterised in detail, it gives a clear indication of failure.

Finally, a caveat to note with this circuit is that we would expect the post-processing variability to be greater than the CMOS variability. As such, we would expect that each LED in the probe would need to be tested and calibrated. But this can be part of the standard infant mortality tests for such devices.

### B. Pulse Width Modulated DAC

This pulse width modulated DAC is based on an adapted switch-capacitor topology, as shown in Figure 6(c). This pulse modulated DAC requires only one digital input signal, and this signal contains the predefined pulse width information. The pulse signal is then combined with a mode selection signal (either *Reset_Pulse* or *Set_Pulse*) to determine the operation mode.

**Fig. 7.**
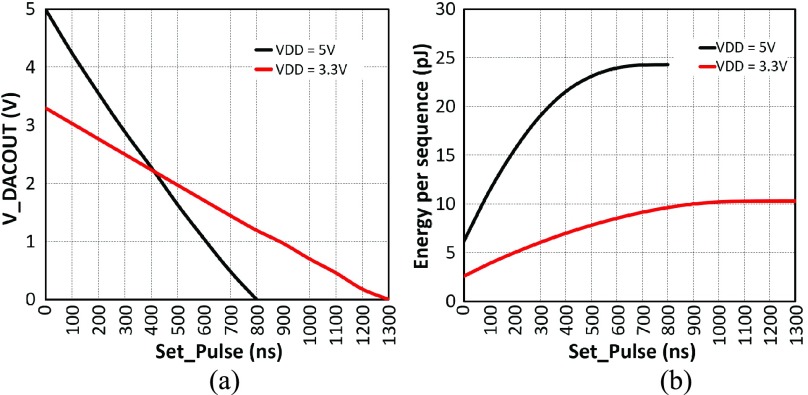
(a) Relationship between *Set_Pulse* and $V_{DACOUT}$. When $V_{DD}$ is 5 V, $V_{DACOUT}$ is gradually decreased from 5 V to 0 V when *Set_Pulse* is swept from 0 ns to 800 ns. When $V_{DD}$ is reduced to 3.3 V, the maximum value of *Set_Pulse* is increased to 1300 ns. (b) Energy consumption analysis per sequence. $V_{DD}$ is set to 5 V and 3.3 V separately. The *Reset_Pulse* is still with the $1~\mu \text{s}$ setting, and *Set_Pulse* signals are tuned in the 0 - 800 ns and 0 - 1300 ns ranges respectively. The maximum energy consumption per sequence is below 25 pJ. If needed, further reduction can be obtained by trimming the *Reset_Pulse* to 300 ns (the minimum charging time for $M_{Cap}$).

If enabling the reset mode, the $M_{1}$ will be closed and $M_{2}$ will be kept open, and then a charge current would drop from $V_{DD}$ to the capacitor. Consequently, $V_{DACOUT}$ will be charged up to $V_{DD}$. If the set mode is selected, $M_{1}$ will be in the open state, and $V_{DD}$ is isolated from the capacitor. At the same time, the $M_{2}$ path will be clear, and $V_{DACOUT}$ will be gradually discharged until the *Set_Pulse* is terminated or $V_{DACOUT}$ is equal to $V_{SS}$. Using this strategy, the value of $V_{DACOUT}$ can be modulated from 0 V to $V_{DD}$ (3.3 V or 5 V), corresponding to the input pulse width. The overall performance of the pulse modulated DAC is illustrated in Figure 7 and then summarized in Table I.

### C. Sigma-Delta ADC

To accomplish the diagnostic circuitry, an ADC module is needed to fetch the diagnostic voltage $V_{Dia}$. Thus, this part presents a low-power miniature $\Sigma \Delta $ ADC, as shown in Figure 6(d). The $\Sigma \Delta $ ADC consists of two components: an *integrator*, and a *reset*. It receives current (4-444 nA) from the sensing amplifier which integrates over a parasitic capacitor $C_{para}$ (18 pF). Once the voltage on the capacitor reaches the threshold voltage on the first inverter stage of the integrator, the output of the $\Sigma \Delta $ ADC goes to $V_{DD}$. This feeds back to the *reset* circuit to discharge the parasitic capacitor, thus forming a pulse. An enable signal on the reset circuit allows for triggering the circuit, and hence either timing between the trigger and the output pulse or the pulse frequency within a given time.

**Fig. 8.**
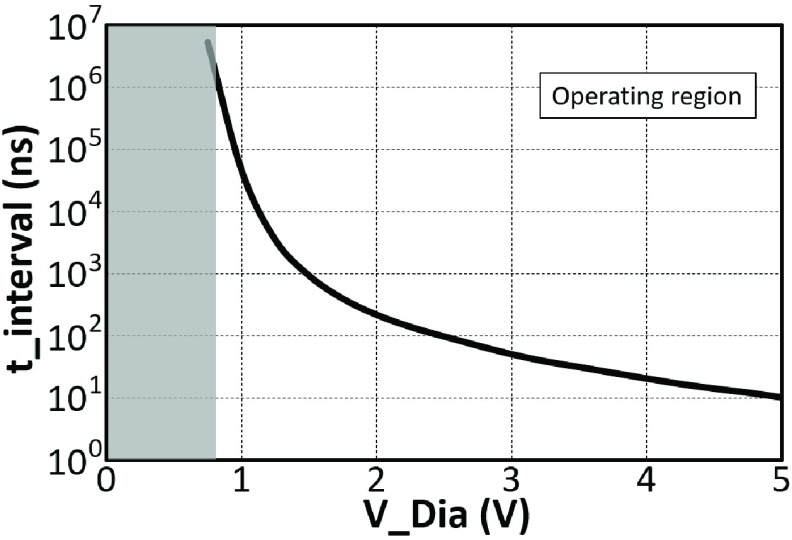
The relationship between time intervals of the output pulse versus the analog input voltage. The $t_{interval}$ has a large dynamic range from nanoseconds to milliseconds, which provides an outstanding sampling resolution.

Given that small transistors were utilised in the integrator, a post-layout simulation was performed to certify its functionality. By tracing this simulation, the correlation between the input voltage and time interval ($t_{interval}$) were deduced subsequently, and the relationship is demonstrated in Figure 8. It can be seen that $t_{interval}$ has a large dynamic range from 10 ns to more than $10^{6}$ ns (> 100 dB). However, as the operational region of the sensing amplifier is $0.75< V_{Dia} <5$ V, the operational dynamic range is $10^{5}$ i.e. 100 dB.

Power analysis is also performed, and the average power consumption has been calculated. When this ADC operates with a 5 V power supply, the power consumption is 12.6 $\mu \text{W}$. When supply voltage decreases to 3.3 V, the power cost is then reduced to $4.8~\mu \text{W}$. Thus, this $\Sigma \Delta $ ADC demonstrates an appropriate power dissipation performance. Similar to the pulse modulated DAC, in actual use, the maximum operation frequency of this $\Sigma \Delta $ ADC is determined by the external controller (FPGA), i.e. 100 MHz in this work. Depending on the requirement, the operation frequency of the $\Sigma \Delta $ ADC can be much lower than 100 MHz. But to ensure appropriate resolution, 5 - 10 MHz clock frequency (dynamic range 70 - 80 dB) would be recommended. Table I summarizes the overall performance of the $\Sigma \Delta $ ADC.

**Fig. 9.**
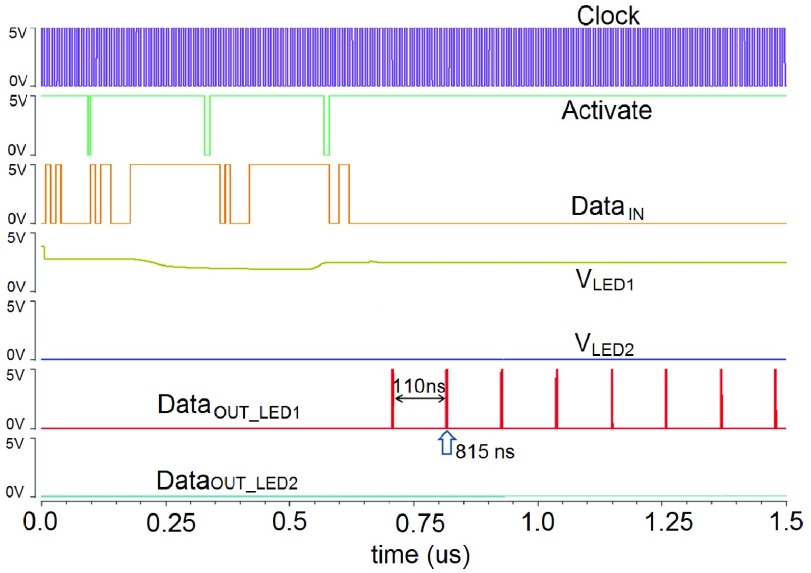
Top-level simulation results for the diagnostic sensing circuitry. A random LED $LED_{1}$ is chosen for analysis, and $LED_{2}$ is selected for comparison. During this simulation, $V_{Sense}$ is set as 3.75 V (e.g. —$V_{gs}$— is equal to 1.25 V), the diagnostic voltage is equal to 2.40 V, and the $t_{interval}$ is correspondingly equal to 110 ns. The local circuit schematic is referred to Figure 6(b).

Then, the diagnostic sensing function is also fully validated via simulations, and Figure 9 illustrates the post-layout simulation results. It can be seen two LEDs (*LED*_1_ & *LED*_2_) are selected to conduct comparative analysis. *LED*_1_ is working in the diagnosis operation state, while *LED*_2_ maintains the off state consistently. The input *Set_Pulse* is defined as 150 ns to accomplish a 3.75 V $V_{Sense}$ signal.

During the simulation, the *Clock* signal defines the operation frequency, and the *Activate* signal controls the diagnostic sensing operation. All of the input information, which can be serially used to achieve system addressing, logic controlling, and pulse signals configuration, are transmitted via *Data* $_{IN}$ signal. In the output part, it can be observed that, after the PWM stage, $V_{LED1}$ ($V_{Dia1}$) is equal to 2.40 V constantly. In contrast, $V_{LED2}$ still maintained at 0.0 V. The second pulse of the *Data* $_{OUT\_{}LED1}$ can be seen at 815 ns, giving a pulse interval of 110 ns. For comparison, no pulsing is seen in *Data* $_{OUT\_{}LED2}$.

In this diagnostic sensing circuit, as mentioned above, the $V_{Sense}$ signal is regulated by the pulse width of the *Set_Pulse*. Then, the accuracy of $V_{Sense}$ modulation relies on the resolution of the pulse width modulated DAC. Likewise, through the $\Sigma \Delta $ ADC, the subsequent $V_{Dia}$ voltage is expressed by a pulse frequency with the time interval $t_{interval}$.

## VI. CMOS Implementation

**Fig. 10.**
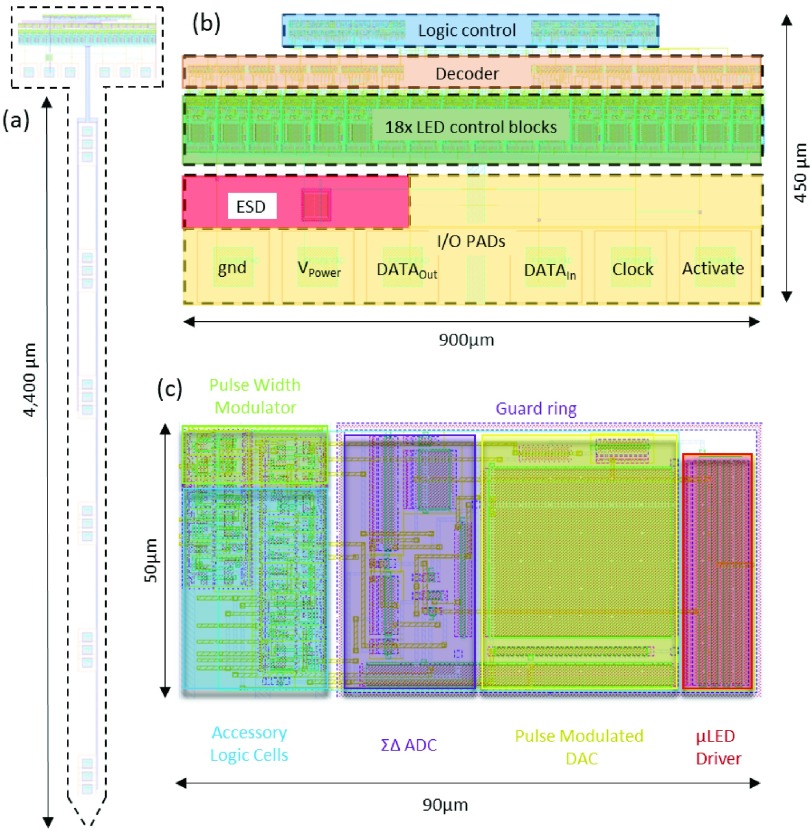
(a) Layout diagram of the optrode. (b) The primary circuitry in the head: six I/O pads are used for power supply and digital & analog I/O, with custom-designed electrostatic discharge (ESD) protection circuitry. The 18 local control blocks consume $90\,\,\mu \text{m}\,\,\times 900\,\,\mu \text{m}$ die area in total. The size of the 18-bit decoder is $20\,\,\mu \text{m}\,\,\times 900\,\,\mu \text{m}$. (c) The layout of integrated local stimulation and diagnostic circuitry. A guard ring is utilized to insulate the analog and digital parts. The area of this local block is only 0.0035 mm^2^.

This optrode has been implemented and fabricated in a X-Fab 0.35-$\mu \text{m}$ 4-metal multi-layer mask wafer process. The global layout and micrograph of the fabricated chip are displayed in Figure 10. The total chip outline on the reticle is $900\,\,\mu \text{m}\,\,\times $ 4850 $\mu \text{m}$, which fits with the proposed architecture in Figure 1. The dimensions of the global logic control block are $35\,\,\mu \text{m}\,\,\times 570\,\,\mu \text{m}$, and the size of the 18 micro-photonic emitter control & sensing blocks is $90\,\,\mu \text{m}\,\,\times 900\,\,\mu \text{m}$. The shaft length is 4400 $\mu \text{m}$, in accordance with the six cortical layers. The micro-photonic emitter anode pad is 60 $\mu \text{m}$. Additionally, the width of metal wirings and spacing is 40 $\mu \text{m}$ on each side. Six micro-photonic emitter clusters are evenly placed along the optrode shaft, and the spacing between each two micro-photonic emitter clusters is 580 $\mu \text{m}$. This should potentially allow for a micro-photonic emitter cluster per cortical layer, though in practice there is a wide variation in cortical layer thicknesses depending on region [26]. In each micro-photonic emitter cluster, only a 20 $\mu \text{m}$ spacing is set between each two micro-photonic emitters. This ensures that those two backup micro-photonic emitters could have a very similar penetration area to that of the main micro-photonic emitter. In addition, the top metal layer metal-L is retained and utilized as a shield for light reflection protection and noise isolation.

## VII. Post-Fabrication

**Fig. 11.**
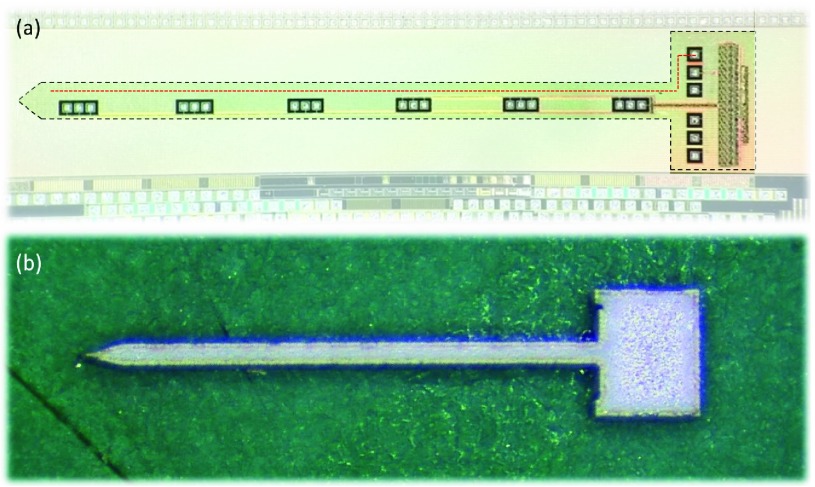
(a) A photograph of the fabricated chip with the primary areas highlighted. (b) An optrode cut out of a (non-CMOS) silicon wafer using a laser ablation method.

A physical layout can be seen in Figure 11(a). The exact cut-out dimensions can be determined according to the user need. Tips can be either blunt or sharp. Sharper tips reduce the required penetration force [37] but increase the potential insertion damage. At a width of $\sim 200~\mu \text{m}$, the probe aspect ratio would be 22, which should be sufficiently low to prevent buckling on insertion.

This work focusses on the circuitry to implement a scalable active optogenetic probe. We, therefore, do not demonstrate full post-fabrication. However, this architecture is compatible with the efforts of Cao [14] and Wu [18]. i.e. a barrier layer, typically titanium or tungsten followed by gold needs to be deposited on the CMOS and LED anode/cathode contact pads. The LEDs need to then be placed on the CMOS optrode and bonded. We would envisage the LEDs being deposited as a single strip to simplify the process. The optrode would then need to be cut out using laser cutting (see Figure 11(b), or a reactive ion etching technique.

Figure 11(a) shows our CMOS device with the optrode dimensions. To demonstrate feasibility, Figure 11(b) shows an exemplar cut-out on normal silicon, using laser-based cutting. All experiments were performed on standard packaged die, as the clean room fabrication technique will be perfected and implemented later.

## VIII. Measurement Results

### A. Experiment Set-Up

The general testing set-up included a Keithley 2602B source meter is utilized to provide the 5 V $V_{DD}$ and the global $V_{SS}$. A Xilinx Virtex-7 FPGA board is selected to configure and control the testing chip package, to support the high-frequency operation of the pulse width modulated DAC. An Agilent 34460A multimeter is used to probe and sense particular analog signals (such as $V_{LED}$, and $V_{Dia}$), while time domain measurement is captured by an Agilent MSO-X 4034A oscilloscope.

Light measurement was achieved by placing a Newport UV-818 calibrated photodiode face-down on an optrode. Currents were then measured using a Keithley source measure unit, and converted to light intensity using the calibration chart provided by Newport. This is the standard method that has been utilized by others [14], [15], [18]. There is, however, a caveat with this approach. We noticed that slight angular torsion of the probe could lead to significantly lower results. Furthermore, even when we achieved optimal alignment, there was some reflection from the photodiode surface, indicating that not all the light was absorbed. As such, we feel our measurements are quite conservative.

### B. Optical Stimulation Function

**Fig. 12.**
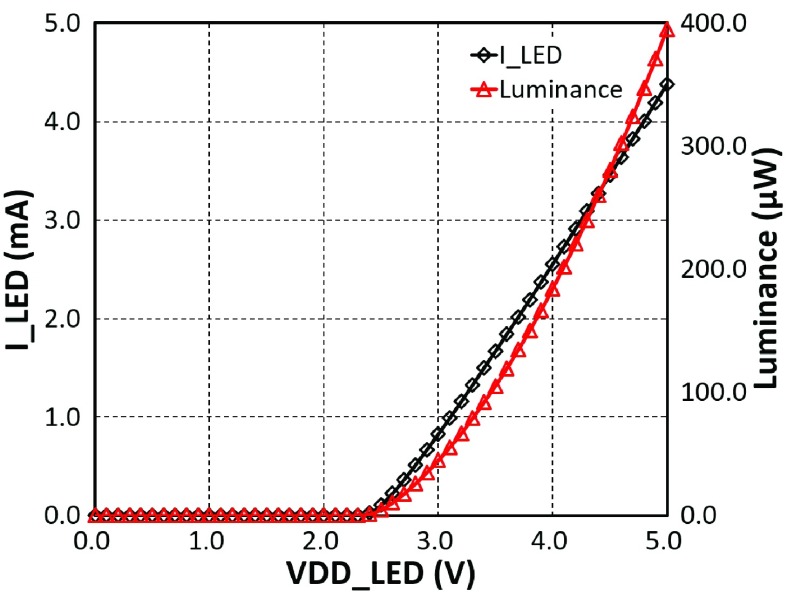
On-bench measurement results for the $\mu $LED current and luminance versus $V_{DD\_{}LED}$. The local circuit schematic is referred to Figure 6(a).

In this part, the optrode stimulation control function has been verified on the bench after setting up the test platform. The input signals (*Clock*, *Activate* and *Data* $_{IN}$) are all generated from the FPGA Virtex-7 board. The primary purpose of the test is to validate the light driving ability of the proposed optrode with the tunable power supply. A solitary $\mu $LED is wire-connected between the LED anode pad and global $V_{SS}$, to measure the drive current. During the measurements, $V_{DD\_{}LED}$ is steadily adjusted from 0.0 V up to 5.0 V with 0.1 V steps. Measurement results are demonstrated in Figure 12. When $V_{DD\_{}LED}$ is lower than the $\mu $LED working threshold, the value of $I_{LED}$ is at the fA or nA level. When $V_{DD\_{}LED}$ is above the threshold, then the light emitter is activated to perform a linear relationship between $V_{DD\_{}LED}$ and $I_{LED}$. It can be seen that, when $V_{DD\_{}LED}$ is equal to 5.0 V, the maximum working $I_{LED}$ is 4.37 mA, and the corresponding effective luminance is 395 $\mu \text{W}$. These results closely match the results shown in Figures 4, 5. This demonstrates that this optrode can generate sufficient luminance with high accuracy, and the targeted light delivery and penetration depth can be achieved.

### C. Diagnostic Sensing Function

The functionality of the diagnostic sensing circuitry has been assessed using the same test platform described above. The sampling points are defined as $\vert V_{gs}\vert = 0.75$, 1, 1.25, and 1.5 V, respectively.

**Fig. 13.**
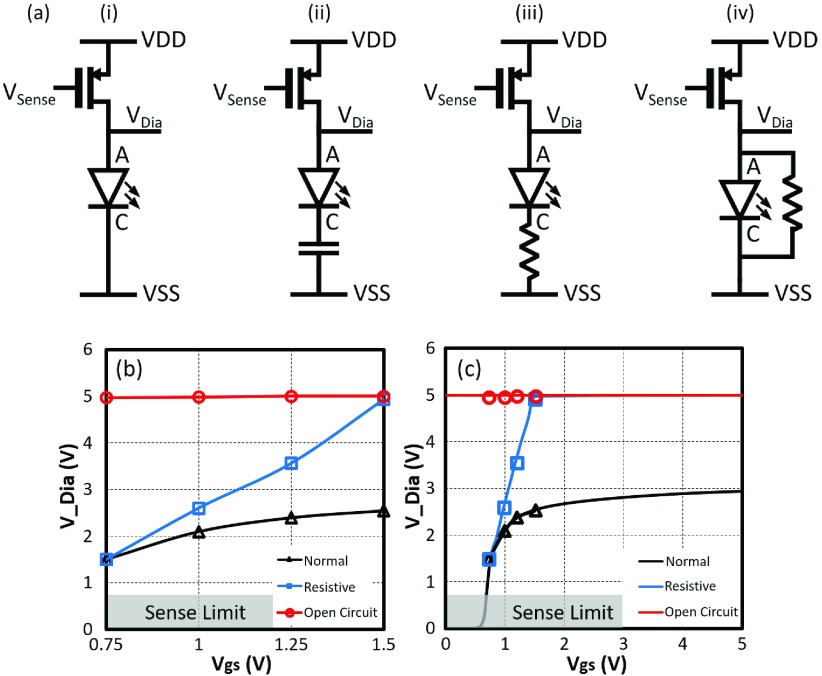
(a) Equivalent four-state circuit model. (b) Comparison measurement results of three-state $\mu $LED operation conditions. (c) Measurement results overlay with the simulation results. The equivalent circuit schematic is referred to Figure 6(b).

To verify the normal operation, a $\mu $LED was connected to the LED anode PAD and global $V_{SS}$. Next, possible degradation mechanisms can be represented as increased resistance or capacitive open circuit. Here, a 500 $\text{K}\Omega $ resistor is picked to act as the increased resistance. It is then serially connected with the $\mu $LED to emulate the corrosion status (resistive state). Meanwhile, to mimic the open circuit, a 10 pF capacitor is connected to the LED PADs. As the resistance of the stimulation site is higher than expected, it can be reflected and extracted by the $V_{Dia}$. The comparison measurement results of the four-state operations are shown in Figure 13. It can be seen from Figure 13(b) that the black curve represents the normal operation: When $V_{gs}$ is in the predefined range, the normal $V_{Dia}$ varies from 1.5 V to 2.5 V. The blue curve demonstrates the corrosion state; with same sampling points, the resistive $V_{Dia}$ is increased from 1.5 V up to 5 V. The red line expresses the optrode rupture status; as an open circuit is formed, the $V_{Dia}$ is equal to 5 V constantly. These measurement results match with the simulation results in Figure 13(c). Therefore, given predefined sampling points, the implant working status can be observed in real-time, and breakage and/or corrosion points will be accordingly detected.

### D. Power Consumption

Based on all the simulation and measurement results previously obtained, the system power consumption of this optrode is calculated and summarized in Table II. It can be seen that the total power consumption of the optrode is 1.008 mW when only one $\mu $LED is turned on at the same time. In particular, the light power consumes the majority of the system power (1.005 mW, 99.7%). In actual use, depending on the battery capacity, up to $18~\mu $LEDs can be simultaneously turned on in the single optrode, or multiple optrodes can be manipulated at the same time if only one specific $\mu $LED needs to be activated.

**TABLE II:** Total Power Consumption of The Proposed Optrode

Subsystem	Major Circuits	Static power ($\mu \text{W}$)	Dynamic power ($\mu \text{W}$)	Utilized circuits duty cycle	Total power ($\mu \text{W}$)
Logic control	FSM	0.00	2	100%	2
Optical Stimulation	Pulse width modulator	0.00	$1.28\times 10^{-3}$	100%	$1.28\times 10^{-3}$
	Logic cells	0.00	$1.06\times 10^{-3}$	100%	$1.06\times 10^{-3}$
	$\mu $ LED	$2.01\times 10^{4}$		5%	1005
Diagnostic sensing	Pulse modulated DAC	$5.77\times 10^{-6}$	8.45	1%	0.0845
	Sensing transistor	100		1%	1
	$\Sigma \Delta $ ADC	$3.3\times 10^{-6}$	12.6	1%	0.126
Total Power ($\mu$W)		1008.21			

## IX. Discussion and Conclusion

This paper has presented a CMOS-based optrode for optoelectronic/optogenetic neural stimulation. The overall system performance of the circuitry in tandem with $\mu $LEDs has been characterized and summarized in Table III.

**TABLE III:** Performance Summary of The Proposed Optrode and Comparison With Other CMOS Active Neural Probes

System Overview				
	This work	[21]	[22]	[23]
Year	2017	2014	2014	2015
Technology	0.35-$\mu \text{m}$, 4-metal X-Fab CMOS process	0.35-$\mu \text{m}$ CMOS process	0.18-$\mu \text{m}$ CMOS process	0.18-$\mu \text{m}$ CMOS process
Operation voltage	5.0 V	3.0 V	1.8 V	1.8 V
Die area	1.36 mm^2^	12.77 mm^2^	10.57 mm^2^	4 mm^2^
Probe head size	$900\,\,\mu \text{m}\,\,\times 450\,\,\mu \text{m}$	-	$3300\,\,\mu \text{m}\,\,\times 2900\,\,\mu \text{m}$	-
Probe shaft size	$4400\,\,\mu \text{m}\,\,\times 200\,\,\mu \text{m}$	-	$10000\,\,\mu \text{m}\,\,\times 100\,\,\mu \text{m}$	$5000\,\,\mu \text{m}\,\,\times 130\,\,\mu \text{m}$
Site area	$20\,\,\mu \text{m}\,\,\times 20\,\,\mu \text{m}$	0.02 mm^2^ (sti)/ 0.035 mm^2^(rec)	$35\,\,\mu \text{m}\,\,\times 35\,\,\mu \text{m}$	$25\,\,\mu \text{m}\,\,\times 25\,\,\mu \text{m}$
No. stimulation sites	18 (scalable to 88) (optical)	64 (electrical)	N/A	N/A
No. recording sites	N/A	256	52 (455 electrodes)	16 (512 electrodes)
Total power	1.01 mW ($1~\mu $LED)/ 6.04 mW ($6~\mu $LEDs)	13.5 mW	1.45 mW	-
Optical Stimulation				
No. $\mu $LEDs	18 (scalable to 88)	N/A	N/A	N/A
$\mu $LED current range	0 - 4.37 mA			
Maximum luminance	$395~\mu \text{W}$			
Modulation methods	Pulse width modulation			
Diagnostic Sensing				
No. sampling points (Min)	4	N/A	N/A	N/A
Sampling rate (Max)	100 MHz			
Input sensing current	0 $-40\,\,\mu \text{A}$			
Output diagnostic voltage	0.75 - 5.0 V			

**Fig. 14.**
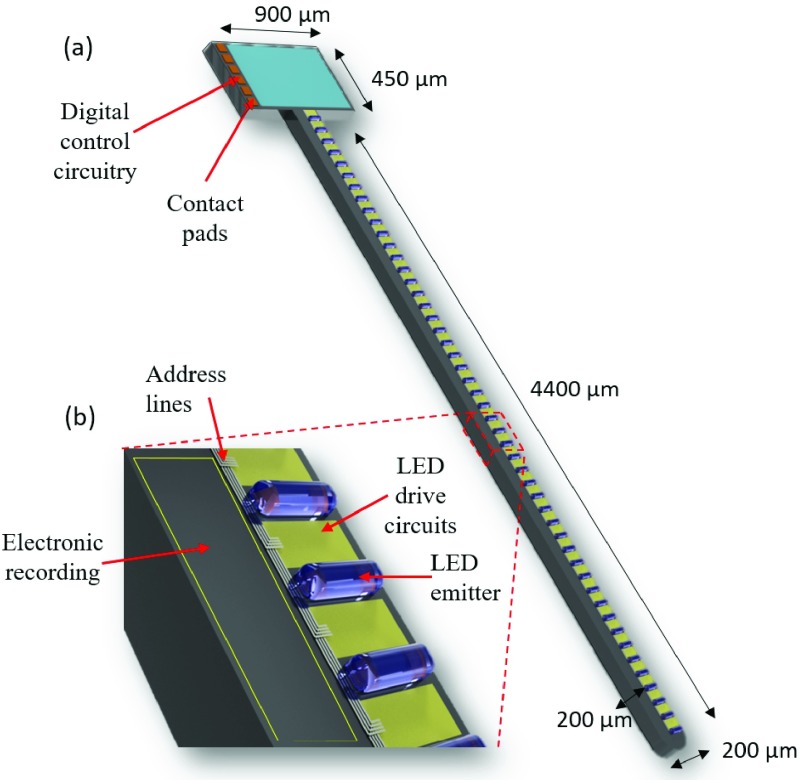
Scalable architecture of the proposed CMOS optrode. The overall size matches with the architecture in Figure 1. Using the outlined dimensions [width $200~\mu \text{m}$, length $4400~\mu \text{m}$, thickness $200~\mu \text{m}$], 49 micro-photonic emitters could be integrated along the shaft. The area outlined in part (b) allows for integration of neural recording circuitry in the future.

An important aspect of the circuitry we have developed is the scalability of the dimensions, as seen in Figure 10(c). If we consider the LED driver as a primary ‘pixel’ with a size of 90 $\mu \text{m}\,\,\times 50\,\,\mu \text{m}$, it could easily be patterned along the shaft, as per Figure 14. Assuming $40~\mu \text{m}$ are required for the micro-photonic emitter bonding, then a repeat unit would be $50+40 = 90 \,\,\mu \text{m}$, allowing for 49 micro-photonic emitters along the optrode shaft. If circuit-under-pad techniques are used, then the repeat spacing could be 50 $\mu \text{m}$, allowing for 88 micro-photonic emitters. Furthermore, scalability includes effectively independent pulse-width and intensity control on each pixel.

Micro-photonic emitter dimensions vary in the literature. We have previously presented devices with total dimensions of 320 $\mu \text{m}\,\,\times 240\,\,\mu \text{m}$ [32], $150\,\,\mu \text{m}\,\,\times 150\,\,\mu \text{m}$ [30] and 80 $\mu \text{m}\,\,\times80\,\,\mu \text{m}$ [13]. McAlinden et al. [15] demonstrated a LED with anode diameter of $40~\mu \text{m}$. Wu et al. [18] demonstrated $15\,\,\mu \text{m}\,\,\times 10\,\,\mu \text{m}$. As such, we feel the proposed dimensions above to be conservative.

Finally, a self-diagnosis sensing scheme has been proposed to assess the optrode integrity and degradation in real-time. To note, this diagnostic circuit can work with both 3.3 V and 5 V voltages, but the LED requires 5 V for high radiance stimulation. Thus, we utilise a 5 V transistor for both stimulation circuit and diagnostic circuit, and mixed circuitry with 3.3 V for non LED-facing transistors will be explored in the future. Though simple, this self-diagnostic function allows for system diagnostics in vivo cases, where it is the only effective way of determining continued effective function of the probe. This would be beneficial for both circuit operation and tissue health.
